# Enhanced anticancer and biological activities of environmentally friendly Ni/Cu-ZnO solid solution nanoparticles

**DOI:** 10.1016/j.heliyon.2024.e39912

**Published:** 2024-10-31

**Authors:** Huma Ayub, Uzma Jabeen, Iqbal Ahmad, Muhammad Aamir, Asad Ullah, Ayesha Mushtaq, Farida Behlil, Binish Javaid, Asad Syed, Abdallah M. Elgorban, Ali H. Bahkali, Rustem Zairov, Asad Ali

**Affiliations:** a Department of Chemistry, Sardar Bahadur Khan Women University, Quetta, Pakistan; b Department of Chemistry, Allama Iqbal Open University, Islamabad, 44000, Pakistan; c Materials Laboratory, Department of Chemistry, Mirpur University of Science and Technology (MUST), Mirpur, 10250, Mirpur, (AJK), Pakistan; d Center for Advanced Studies in Vaccinology & Biotechnology (CASVAB), Quetta, Pakistan; e Department of Biochemistry, Sardar Bahadur Khan Women University, Quetta, Pakistan; f Department of Biotechnology, Mirpur University of Science and Technology (MUST), Mirpur, 10250, Mirpur, (AJK), Pakistan; g Department of Botany and Microbiology, College of Science, King Saud University, P.O. Box 2455, Riyadh, 11451, Saudi Arabia; h Aleksander Butlerov Institute of Chemistry, Kazan Federal University, Kazan, 420008, 1/29 Lobachevskogo str., Russian Federation; i Arbuzov Institute of Organic and Physical Chemistry, Kazan Scientific Center, Russian Academy of Sciences, 8 Arbuzov str., 420088 Kazan, Russian Federation; j Energy Engineering, Division of Energy Science, Luleå University of Technology, 97187, Luleå, Sweden

**Keywords:** ZnO NPs, Solid solutions, Band gap tuning, Anticancer, Antioxidant activities

## Abstract

The study investigates the impact of incorporating Ni and Cu into the lattice of ZnO nanoparticles (NPs) to enhance their anticancer and antioxidant properties. Characterization techniques including pXRD, FTIR, UV–visible absorption spectroscopy, FESEM, and EDAX confirm the successful synthesis and structural modifications of Ni/Cu-ZnO NPs. Anticancer activity against breast cancer (MDA) and normal skin (BHK-21) cells reveals dose-dependent cytotoxicity, with Ni/Cu-ZnO NPs exhibiting higher efficacy against MDA cells while being less harmful to BHK-21 cells. Morphological studies corroborate these findings. Additionally, antioxidant assays using TAC, FRAP, and DPPH assay demonstrate the superior antioxidant activity of Ni/Cu-ZnO NPs matched to pure ZnO. Overall, the synergistic effect of Ni and Cu incorporation leads to improved therapeutic potential, making Ni/Cu-ZnO NPs promising candidates for cancer therapy and antioxidant applications.

Molecular docking recreations were performed using Auto Dock Vina software to gain more insights and validate the observed biological activities of un-doped ZnO and bi-metal doped ZnO NPs, we investigated the interaction and binding affinities of pure ZnO and bimetallic metal co-doped ZnO for their antioxidant and anticancer studies. Ni/Cu-ZnO have shown good antioxidants and exhibited remarkable anticancer activities.

## 1 Introduction

**Scheme 1(1, 2):**
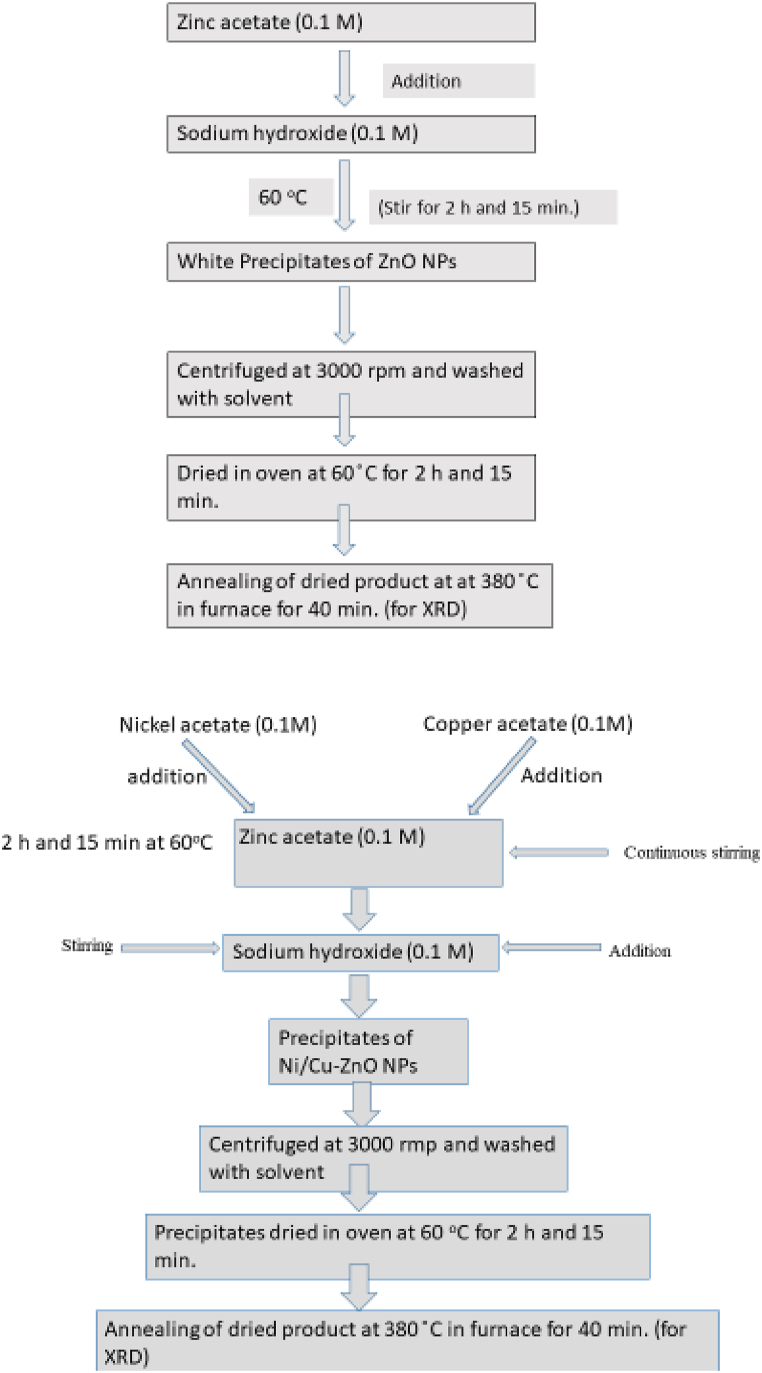
The schematic representation to produce un-doped and Ni/Cu-ZnO NPs.

Cancer is one of the frequent causes of death worldwide [1]. Several limitations such as nonspecific toxicity, drug resistance, and high doses have been associated with chemotherapy [2]. Therefore, safe and effective treatment of cancer has become an area of focus for the scientific community. Nanomaterials offer alternative cancer therapy methods [3,4]. Among various nanomaterials, the environmentally friendly ZnO nanoparticles have gained enormous attention due to their tuneable physiochemical features. Besides the physiochemical features, ZnO NPs exhibit particular cytotoxicity towards cancerous cells while keeping healthy cells unaffected. For example, Researcher have observed the effect of ZnO NPs effects the human glioma cells [5]. The BHK-21 cell line has been extensively studied for various purposes, including virological research and quality control of biological products [6]. Copper posses remarkable characteristics such as high electrical conductivity, ductility and thermal property [7]. Copper is a necessary mineral for living organs due to the pivotal contribution to the formation of the respiratory enzyme cytochrome oxidase C [8]. Copper NPs exhibit unique properties such as antibacterial activity [9] and catalytic, antifungal [10], and Commercially available copper does not exhibit these properties. CuO NPs with apoptotic effects on cancer cells are promising anticancer agents [11]. After iron, zinc is the rarest trace mineral with highest quantities in the body [12]. Zinc Finger Proteins and enzymes like Superoxide Dismutase contain zinc [13,14]. Studies shows an immediate relationship between the body's zinc levels and cellular inflammation. Immune cells are stimulated when the body contains less zinc. In this sense, the body's cells become inflamed due to a zinc deficiency. The body's severe negative effects of zinc shortage include some cells becoming inflamed and cancerous cells being stimulated [15,16]. High quantities of zinc cause cancer cells to undergo apoptosis, research has demonstrated the importance of zinc as a therapeutic agent in the prevention and treatment of cancer. It is frequently used in cosmetics and is also referred to as a skin and tissue repairer [17,18]. Numerous transition metals and their oxides have been investigated as electrode substrates for glucose oxidation [19]. Among these materials, Nickel oxide NPs have gained appeal to lately because of their remarkable catalytic activity and electrochemical durability [19]. Furthermore, the use of environmentally friendly methods like green synthesis, which involves non-toxic and biocompatible approaches, is encouraged for nanoparticle synthesis [20]. These detections collectively highlight the significance of assessing the biocompatibility of Ni/Cu-ZnO NPs synthesized through environmentally friendly methods to ensure their safety and efficacy in biomedical applications. Research reported that the ZnO NPs specifically kill the liver (HepG2) and human lung (A549) cancer cells. However, primary rat astrocytes and hepatocytes remained unaffected [21]. ZnO NPs have recently been demonstrated in an orthotropic mice model to demonstrate anticancer properties against human small cell lung cancer [22]. However, still, many limitations have been associated with the development of anticancer agent-based ZnO NPs. The emergence of various new cancers and pathogenic species that cause infections and severe diseases, has directed to improve the functionalities of the ZnO NPs to mitigate such challenges (see Scheme 1).

It was found that the anticancer performance of the ZnO NPs can be intensified by metal ions co-doping the ZnO NPs [23]. The antibacterial, anticancer activities, and antimicrobial of the ZnO NPs have been improved by doping with transition metal ions [23,24]. In anticancer activities, the Ag-doped ZnO has shown a promising result compared to the pure ZnO NPs. Likewise, the rare earth metal ions doped with undoped ZnO NPs have been known as potent biomarkers for early cancer diagnosis [25,26]. However, the precious metal and rare earth metal doping is not a cost-effective approach. Transition metals such as Ni, Cu, Mn, Fe, etc. are being used as a prominent doping agent in ZnO lattices [27,28]. The co-doping of ZnO lattice with the transition metal ions effectively improves the optoelectronic properties as well as the biological performance.

Our research focuses on a novel approach for synthesizing Ni/Cu-ZnO using the co-precipitation method. Through comprehensive characterization techniques including X-ray diffraction (XRD), scanning electron microscopy (SEM), energy dispersive X-ray spectroscopy (EDX), Fourier-transformed infrared spectroscopy (FTIR), and UV–Vis absorption spectroscopy, we examined the synthesized samples. This study marks the first investigation into the carceno preventive properties of Ni/Cu-ZnO, evaluated via the MTT method on epithelial breast cancer cells MDA and skin cell BHK-21. Our findings unveil promising biological applications of these nanocomposites. Future research avenues could delve deeper into the cytotoxic effects of Ni/Cu-ZnO on cancer cells, elucidating their mechanisms of action and potential for targeted cancer therapy. This study fills a crucial gap in current investigate.

Moreover, assessing the biocompatibility of Ni/Cu-ZnO NPs is authoritative for their suitability in biomedical applications. Expanding on this aspect will further validate their potential in medical contexts.

## 2 Experimental

### 2.1 Chemical reagents

Zinc acetate dihydrate (Zn(CO_2_CH_3_)_2_.2H_2_O), copper acetate (Cu(CO_2_CH_3_)_2_), sodium hydroxide (NaOH), and acetone C_3_H_6_O were utilized for the synthesis of un-doped and Ni/Cu doped ZnO of various concentration. Sigma Aldrich provide all chemicals used in the research.

### 2.2 Synthesis of ZnO nanoparticles

Zinc Oxide Nanoparticles (ZnO NPs) were synthesized employing the co-precipitation technique. A mixture of 100 mL of zinc acetate (0.1 M) and 100 mL of sodium hydroxide (0.1 M) was prepared in a beaker, followed by stirring solid solution at 60 °C for 2 h and 15 min while retaining continuous agitation to manufacture white ZnO NPs precipitates. The resultant solid product underwent centrifugation at 3000 rpm to isolate the precipitates, which were subsequently washed twice with water and once with acetone. Finally, material was dried at 60 °C for 2 h and 15 min in an oven.

### 2.3 Synthesis of Ni/Cu-ZnO nanoparticles

For 10 % Ni/Cu-ZnO NPs fabrication, 10 mL of (0.1M) nickel acetate solution and (0.1M) copper acetate and 80 mL of (0.1 M) zinc acetate were mixed with a dropwise addition of 80 mL of (0.1M) sodium hydroxide under constant stirring for 2 h and 15 min at 60 °C. White precipitates of Ni_10_Cu_10_ZnO_80_ NPs were obtained. Precipitates were centrifuged and washed with distilled H_2_O twice and once through acetone and centrifuged at 3000 rpm. Now dry the precipitate at 60 ^°^C in the oven. For the (20 %) Zn_60_Ni_20_CuO_20_ and (30 %) Zn_40_Ni_30_CuO_30_ same procedure was repeated by just adding the suitable concentration (x = 0.1M, 20 mL each), (y = 0.1 M, 30 mL each) of nickel and copper precursors.

## 3 Anticancer activity

### 3.1 Cell culture

In this study, MDA and BHK-21 cells were employed to estimate the cytotoxicity of synthesized samples. Initially, the cells were thawed and removed for cultivation. Following this, they were placed in Falcon tubes and centrifuged for 10 min at 1500 rpm. After the upper liquid layer was separated, the cells were resuspended in an enriched culture medium. The cell cultivation process required DMEM culture medium enriched with 100 μg/mL streptomycin, 100 international units/mL penicillin, and 10 % fetal bovine serum (FBS) to mitigate microbial contamination. To facilitate cell proliferation and growth, the culture medium was subjected to incubation in an environment containing 5 % CO_2_ at 37 °C. Each well of the 96-well plate undergoes 24 h incubation process. Upon verifying the adherence of the cells to the underside of the plate, the medium supporting cell growth was aspirated from each well of the 96-well plate. Subsequently, 100 μL of nanoparticles (NPs) were introduced into the designated wells, after which the plate was placed in an incubator set at 37 °C with 5 % CO2 for 24 h.

### 3.2 MTT assay

The % viability and % toxicity of MDA epithelial breast cancer cells and BHK-21 kidney skin cells were determined using the MTT assay after exposure to un-doped and Ni/Cu-ZnO, hence without treatment (control) [29,30]. For this experiment, the BHK-21 and MDA cell lines were first seeded for 24 h at 37 °C in a moist environment in specialized 96-well plates (at a rate of 1 × 10^4^ and 5 × 10^3^/well), respectively. The cells were then treated for 24 h with un-doped and Ni/Cu-ZnO nanoparticles at different concentration controls, 30, 60, 120, and 240 μg/mL.

After the cancer cells in the plates were thoroughly mixed, a 100 μL portion of the cellular suspension was mixed with (5 mg/mL in phosphate-buffered saline) in a proportion of 10 μL per well and a pre-prepared concentrated solution of MTT assay. The plates then went to a 4-h incubation period. After the incubation process proceeded well, the solution was detached from the wells and mixed gently with 200 μL of DMSO to dissolve the Farmazan product. At 550 nm, the solution's optical characteristics were evaluated using a microplate reader (Multiskan Ex, Thermo Scientific, Vantaa, Finland). Under the same conditions, control cells were tested and used as a reference. This equation was employed for the computational of cell viability.

$$\%\;Viability=\left[\frac{\left(mean\;absorbance\;of\;treated\;group\right)}{\left(mean\;absorbance\;of\;control\;group\right)}\right]\times 100$$

## 4 Antioxidant studies

### 4.1 Total antioxidant activity (TAC)

The total antioxidant activity (TAC) was revealed using the phosphor-molybdenum methodology (Sadhu, Okuyama, Fujimoto, & Ishibashi, 2003). Un-doped and 20 % Ni/Cu-ZnO were produced dilutions of 30, 60, 120, 250, and 500 μg/mL 5 mL of a solution containing (0.6 M H_2_SO_4_, 28 mM Na_2_SO_4,_ and 4 mM (NH_4_)6Mo_7_O_24_ 4H_2_O, and are added to 0.5 mL of each transition metal co-doped zinc oxide solution. At 95 °C, the test tubes completed an incubation process. After reaching room temperature, a UV/Visible spectrophotometer was employed for the determination of absorbance at 695 nm. The same procedure is applied to the blank when methanol is used in place of the NPs solution. The TAC of the samples under analysis was determined by utilizing a specific equation.

$$TAC\;\%=\left[\;\frac{\left(A_{o}-A_{t}\right)}{A_{o}}\right]\times 100$$

where A_t_ is the tested sample's absorbance value and A_o_ is the absorbance of the control sample, specified time. The reference standard used was ascorbic acid. The results (TAC) of the linear regression calculation are shown as the ascorbic acid equivalents in grams per milliliter. Each experiment was executed three times, and the average absorbance for each concentration was recorded [31].

### 4.2 DPPH method

ZnO NPs ability to scavenge free radicals was tested using the DPPH approach [32]. A volume of 1 mL (mL) of the solution containing zinc oxide co-doped with a transition metal was mixed with two milli liters (mL) of DPPH (1,1-diphenyl-2-picrylhydrazyl) (0.004 %) dissolved in methanol. The concentrations of undoped zinc oxide (ZnO) and transition metal doped ZnO nanoparticles (NPs) varied from 30, 60, 120, 250, to 500 μg/mL. Subsequently, this combination was incubated for 1 h. The absorption measurement was then conducted by UV/VIS spectrophotometer at a wavelength of 517 nm. Methanol assisted as a blank solution in this process. Methanol and a 1:1 mixture of DPPH was utilized as the experimental controls. Ascorbic acid was employed as the positive control compound in the study. A specific mathematical equation was utilized to calculate the level of inhibition, which was expressed as a percentage:

$$\%\;Inhibition=\left[\;\frac{\left(A_{c}-A_{e}\right)}{A_{c}}\right]\times 100$$

where; A_c_ and A_e_ are the absorbance of the NPs and control, respectively.

### 4.3 Ferric reducing antioxidant power (FRAP)

The antioxidant activity of Ni/Cu-ZnO nanoparticles was assessed using the method outlined in Ref. [33]. The FRAP reagents was prepared by mixing the following reagent in a ratio of 10:1:1 (vol/vol): acetate buffer (8 mL of acetic acid, 1.6 g sodium acetate and made volume up to 500 mL by DI water), pH 3.6, 2,4,6-tripyridyl-s-triazine (TPTZ) 10 mM solution in 40 mM HCl and FeCl_3_ 20 Mm solution. At 38 °C, the freshly made FRAP reagent was warmed. The bimetallic transition metal oxide NPs comprising 0.5 mL were combined with 4 mL of the FRAP reagent and gently mixed. Well with the assistance of an Ultraviolet Visible (UV/VIS) spectrophotometer, the measurement of absorbance at 593 nm was conducted. Following the same protocol, an ascorbic acid standard curve was generated, mg ascorbic acid/g of sample extract is the way the results were reported.

### 4.4 Determination of reducing power (RP)

The typical method was used to calculate the RP of un-doped and Ni/Cu-ZnO [33]. The transition metal co-doped ZnO of various concentrations was treated with 2.5 mL potassium ferricyanide K_3_[Fe(CN)_6_] and 2.5 mL phosphate buffer in methanol. The blend was incubated at a temperature of 50 °C for 30 min. Following cooling to room temperature, a volume of 2.5 mL of 10 % trichloroacetic acid was introduced into the mixture, which was subsequently subjected to centrifugation at 3000 rpm for 5–10 min. Three mL of the upper layer of the solution, one mL of freshly made ferric chloride (0.1 %) solution, and three mL of distilled H_2_O were combined. Using a UV/VIS spectrophotometer, then the absorbance was quantified at wavelength of 700 nm. Ascorbic acid was utilized in various quantities as a standard.

### 4.5 Molecular docking analysis

The molecular docking analysis was executed using AutoDock Vina software [34,35]. The protein structures were obtained from the Protein Data Bank and prepared using the Discovery studio visualizer, which involved removing water molecules and co-crystallized ligands, while the molecular docking study was carried out using AutoDock Vina. Protein structures were optimized with the default parameters, and a grid box was used to specialize the binding pocket.

**Fig. 1:**
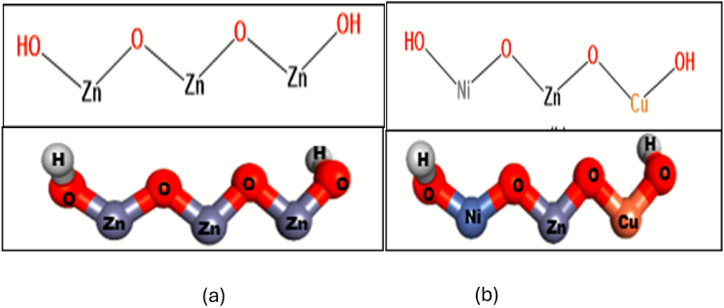
2D and 3D structure of (a) Undoped ZnO (b) Ni/Cu doped ZnO.

As seen in Fig. 1(a) and (b), the crystal structure of ZnO was modified to generate the structure of undoped ZnO and Ni/Cu-ZnO NPs. ZnO was obtained from PubChem in CIF format and processed using Gaussian 09 software. For every target protein, ten best-docked conformations were produced to obtain insight into the patterns of interaction between NPs and active site residues. Ni/Cu-ZnO is a crystal made up of repeating unit cells stacked in a regular array. The fundamental monomeric structure of Ni/Cu-ZnO was utilized as a representation of the complete crystal structure to assess different potential interactions with active site residues. It is crucial to take into account the natural environment of the chosen protein targets in in silico predictions, which includes solvents, ions, and other factors. As monomeric units, the NPs enter the active pocket and engage in electrostatic, hydrophobic, and van der Waals interactions with different residues. Since it is not possible to represent the interaction pattern at the atomistic level using the entire crystal structure, the monomeric units are used instead.

### 4.6 Antioxidant study

**Fig. 2:**
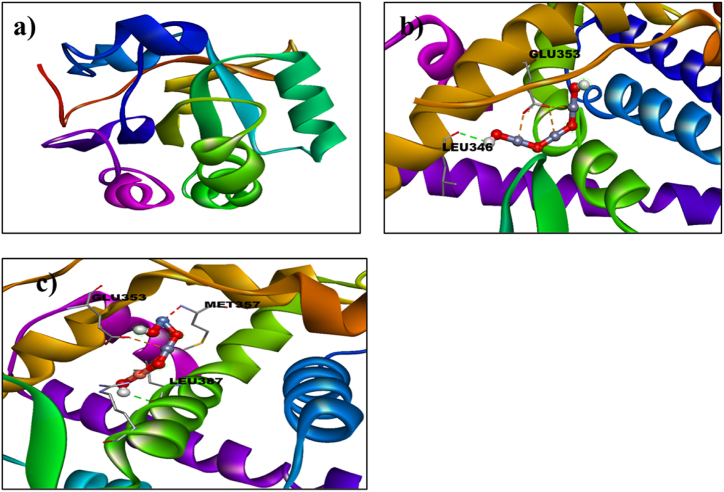
(a): 3D Structure of human peroxiredoxin 5 (PDB ID: 1HD2), (b) Binding interaction pattern of un-doped ZnO, (c) Ni/Cu-ZnO NPs with the active pocket of human peroxiredoxin 5 (3D-view).

A molecular docking research was conducted to assess the antioxidant activity of undoped ZnO and Ni-Cu/ZnO in the human peroxiredoxin 5 (PDB code 1HD2) active site. Human peroxiredoxin 5 (PRDX5) is a key enzyme that directly neutralizes H_2_O_2_ and other ROS. It plays roles in cell processes and oxidative stress defense like differentiation and apoptosis and may have broader ROS activity than other similar enzymes [36]. The 3D structure of the human peroxiredoxin 5 (PDB code 1HD2: Resolution: 1.50 Å) was obtained from the protein data bank as appeared in Fig. 2(a) [37].

### 4.7 Anticancer study

**Fig. 3:**
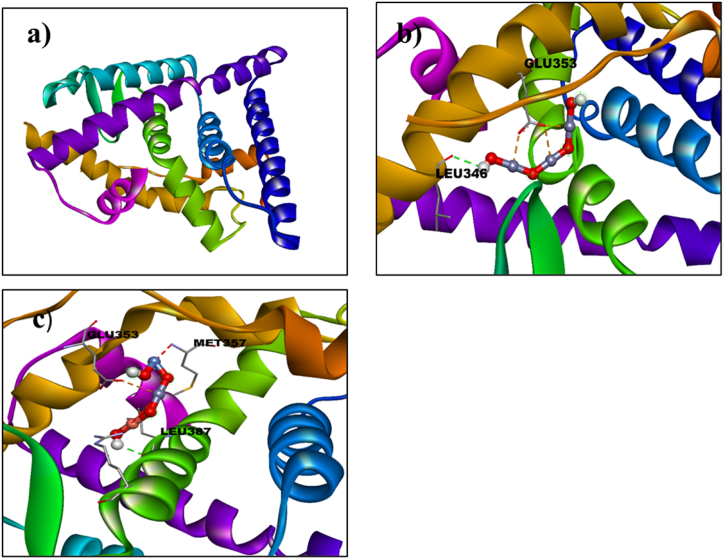
a) 3D Configuration of estrogen receptor α (ERα) (PDB ID: 5GS4, (b) Binding interaction pattern of un-doped ZnO, (c) Ni_20_Cu_20_ZnO_60_ NPs with the active pocket of estrogen receptor α (3D-view).

The binding interactions of undoped ZnO and Ni/Cu-ZnO with potential breast cancer targets were assessed through a molecular docking study. The estrogen receptor alpha (ERα; PDB: 5GS4) was chosen as the protein target for this analysis. ERα is the primary clinical biomarker for subtyping breast cancers and is essential in the development and progression of hormone-dependent breast cancer, making it a key target for this research [38]. The 3D structure of estrogen receptor alpha (ERα; PDB: 5GS4) resolution: 2.40 Å [39] was obtained from the protein data bank Fig. 3(a).

## 5 Molecular docking of BHK-21 for antioxidant and anticancer activity

### 5.1 Antioxidant study

**Fig. 4:**
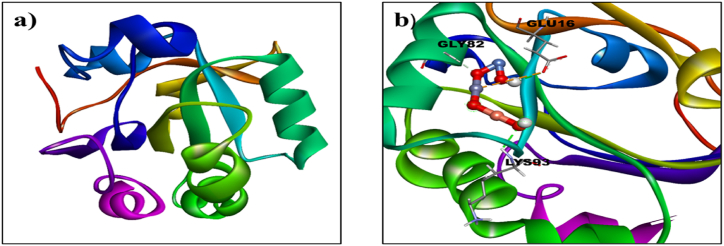
(a) 3D Configuration of human peroxiredoxin 5 (PDB ID: 1HD2), (b) Binding interaction types of Ni/Cu-ZnO with the active pocket of human peroxiredoxin 5 (3D-view).

The un-doped ZnO and Ni_20_C_20_ZnO_60_ underwent a molecular docking analysis to assess their antioxidant capabilities within the active site of human peroxiredoxin 5 (PDB code 1HD2: Resolution: 1.50 Å). Human peroxiredoxin 5 (PRDX5) functions as a crucial enzyme that directly counteracts Reactive oxygen species (ROS), such as H_2_O_2_, which play roles in cellular processes including differentiation and apoptosis oxidative stress response and potentially exhibit broader ROS activity compared to analogous enzymes [36]. The three-dimensional configuration of human peroxiredoxin 5 (PDB code 1HD2: Resolution: 1.50 Å) was obtained from the protein data bank Fig. 4(a) [37].

**Fig. 5:**
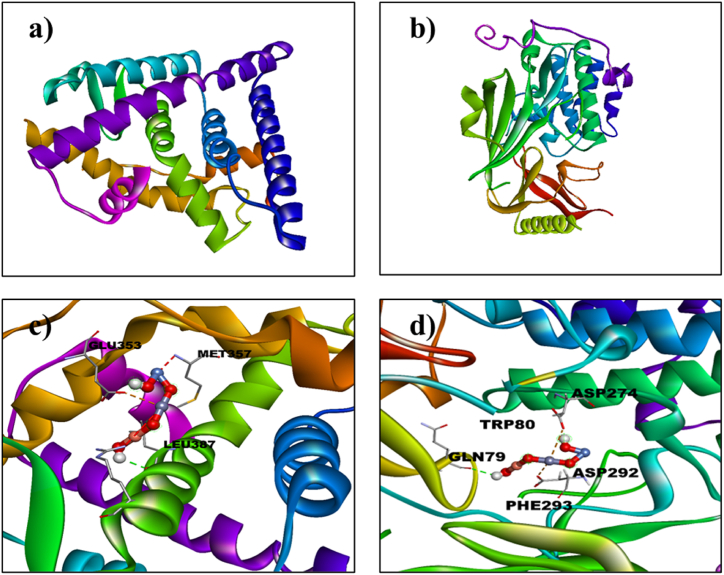
(a) 3D Structure of estrogen receptor α (ERα) (PDB ID: 5GS4), (b) 3D structure of AKT1 target proteins (PDB ID:7NH5), (c) 3D depiction of the binding contact pattern between Ni_20_Cu_20_ZnO_60_ with the active pocket of estrogen receptor α, (d) Binding contact pattern of Ni_20_Cu_20_ ZnO_60_ with the active pocket of Akt1 target (3D-visualization).

The undoped ZnO and Ni/Cu incorporated in ZnO were exposed to a molecular docking study to evaluate their binding arrangement of interactions with potential points for anticancer activity. Two important protein targets i.e. The protein kinase Akt target [PDB:7NH5] for BHK-21 normal skin cell lines and estrogen receptor alpha (ERα; PDB: 5GS4) for breast cell lines were selected for the molecular docking analysis respectively. This study focused on evaluating the binding interactions of undoped ZnO and Ni/Cu-ZnO NPs with potential anticancer targets, Estrogen receptor alpha (ERα) is the primary clinical biomarker used for subtyping breast tumors. Especially significant is its role in the improvement and development of hormone-dependent breast cancer [38]. Moreover, Akt1, a Protein Kinase, serves as a crucial regulator of cell survival, metabolism, and proliferation. The 3D configuration of estrogen receptor alpha (ERα) with a resolution of 2.40 Å (PDB: 5GS4) is depicted in Fig. 5(a) [39], Meanwhile, the 3D crystal structure of Akt1 (PDB: 7NH5) at a resolution of 1.9 Å was acquired from the protein data bank, as depicted in Fig. 5 (b) [40].

### 5.2 Characterizations

A UV–Vis spectrophotometer (Shimadzu 1601) was employed for the investigation of the optical characteristics of both undoped and Ni/Cu co-doped ZnO nanoparticles. Utilizing scanning electron microscopy (SEM) (MIRA3 TESCAN) in conjunction with Energy Dispersive X-ray spectroscopy (EDAX) was crucial in determining the configuration, arrangement, and dimensions of the synthesized NPs. The assessment of the crystallographic structure and phase purity of both undoped and bi-metal co-doped zinc oxide NPs was performed through powdered X-ray diffraction (p-XRD) measurements. Fourier transform infrared spectroscopy (FTIR) spectra were acquired using an FTIR spectrometer (Nicolet 6700) in the ATR mode.

## 6 Results and discussion

**Fig. 6:**
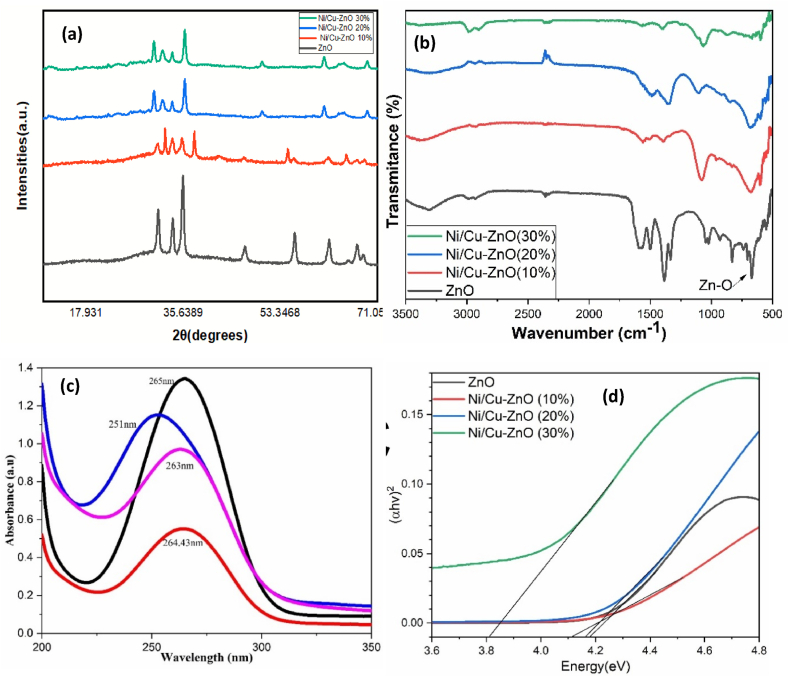
(a) pXRD spectra, (b) FTIR spectra, (c) absorption spectra, and (d) band gap of pure ZnO, Ni_10_Cu_10_ZnO, Ni_20_Cu_20_ZnO, and Ni_30_Cu_30_ZnO nanoparticles.

pXRD spectra of undoped ZnO and Ni/Cu incorporated ZnO (10 %, 20 %, and 30 %) solid solution are shown in Fig. 6(a). The XRD spectra for pure ZnO show peaks at 2θ which were indexed to (100), (002), (101), (102), (110), (103), (200), (112), (201) of pure hexagonal wurtzite ZnO appearance which are in good agreement with the JCPDS: 89-1397 [41]. Three preferred orientation peaks corresponding to the lattice plane (100), (002), and (101) are observed in all samples. The unreacted NiO or CuO or other extra impurities may create the secondary phases which was not observed in all the as-synthesized samples. But an additional peak at about 40 Fig. 6 (a), is observable for 10 % nickel and copper zinc oxide NPs due to nano size or due to Ni(OH)_3_ secondary phase examined and reported by Pal [42]. pXRD peaks did not shift upon nickel and copper incorporation, showing that no massive crystalline distortion of the crystal lattice happened upon nickel and copper concentration increase in the ZnO lattice. The introduction of dopants into ZnO modifies the lattice parameter, impacting its diffusion across the crystal structure, both Ni and Cu replaced the zinc site in the ZnO. The crystallite size for Zn_80_Ni_10_Cu_10_O, Zn_60_Ni_20_Cu_20_O, Zn_40_Ni_30_Cu_30_O were 9.6, 10.3, and 13.6 nm and ZnO was 21.4 nm [43].

FTIR spectra of as-prepared samples have appeared in Fig. 6(b). The band at ∼3380 cm^−1^ represents the stretching vibrations for the -OH bond. A peak at ∼2300 cm^−1^ confirms the existence of atmospheric CO_2_ [44]. A stretching vibration for the Zn-O bond was perceived at 500-600 cm^−1^. As the concentration of Ni and Cu increased from 10 to 30 %, the stretching vibration band for the metal oxide bond is shifted to ∼800 cm^−1^. Observing a rise in vibration frequencies implies that Ni+2 and Cu+2 ions have been incorporated into the hexagonal wurtzite structure.

At room temperature, the optical characteristics of synthesized zinc oxide-based solid solutions were examined using UV–Visible absorption spectroscopy as shown in Fig. 6(c). The absorption maxima observed at 265, 264, 251, and 263 nm for pure ZnO, Ni_10_Cu_10_ZnO_80_, Ni_20_Cu_20_ZnO_60_, and Ni_30_Cu_30_ZnO_40_, respectively Blue shift was observed in absorption spectra of un-doped and co-doped ZnO. The monodisperse distribution of nanoparticles was indicated by the significant sharp absorption peaks [45]. In comparison to other transition metals doped ZnO, Cu-ZnO exhibited a sharp band edge which could be attributed to the accumulation of defect states near the conduction band [45,46]. The blue emission is because of the charge transfer between Cu^2+^ ions and neighboring oxygen atoms, the blue band peak intensities were increased as the copper doping concentration increased [47].

Band gap energies for ZnO and Ni/Cu-ZnO were determined through the utilization of tauc's plot. Observations revealed a direct decrease in the band gap energies of fabricated samples, ranging from 3.96 to 3.84 eV for pure ZnO, Ni_10_Cu_10_ZnO_80_, Ni_20_Cu_20_ZnO_60_, and Ni_30_Cu_30_ZnO_40_ nanoparticles (Fig. 6(d)). This decline in band gap energy is directly linked to Ni^2+^ and Cu^+2^ ions in the ZnO structure. The introduction of these metallic ions within the ZnO lattice leads to the creation of energy levels in close nearness to the conduction band, consequently resulting in a reduction of its band gap. The simultaneous increase in particle size and decrease in band gap can be interpreted as a manifestation of the quantum confinement effect [48].

**Fig. 7:**
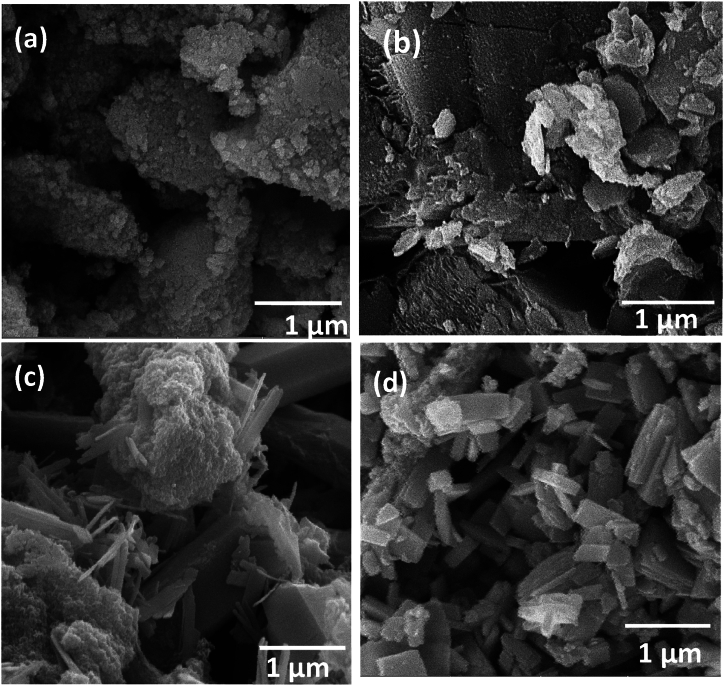
SEM Micrographs of (a) pure ZnO, (b) Ni_10_Cu_10_-ZnO_80_ NPs (10 %), (c) Ni_20_Cu_20_-ZnO_60_ NPs (20 %), (d) Ni_30_Cu_30_-ZnO_40_ NPs (30 %).

Fig. 7(a–d) signifies the FESEM images of the pure ZnO and Ni_10_Cu_10_ZnO, Ni_20_Cu_20_ZnO, and Ni_30_Cu_30_ZnO nanoparticles. Pure ZnO is found to have smaller spherical particles agglomerated into larger spheres as shown in Fig. 7(a). No surfactant was used during the synthesis process so it can efficiently remove aggregation of nano crystals [42,49]. The incorporation of Ni and Cu into the ZnO lattice alters the morphology of particles to rectangular and needle-shaped structures Fig. 7(b–d). With an increase in concentration of Ni and Cu incorporation into ZnO, rectangular morphology is attained.

**Fig. 8:**
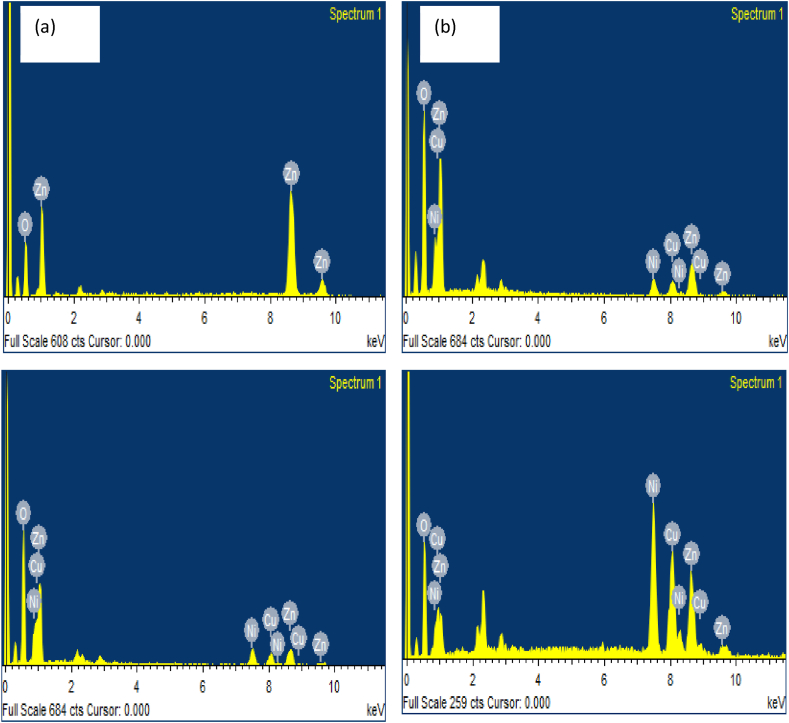
The EDAX spectra of (a) ZnO NPs (b) Ni_10_Cu_10_ZnO_80_ NPs (10 %), (c) Ni_20_Cu_20_ ZnO_60_ NPs (20 %), (d) Ni_30_Cu_30_ZnO_40_ NPs (30 %).

Energy dispersive X-ray spectroscopy was used to explore the chemical constitution of samples that we produced. Fig. 8(a–d) shows the EDAX spectra of the ZnO and Ni/Cu-ZnO NPs. All the spectra confirm the purity of the prepared samples. As shown in Fig. 8(a), the presence of Zn and O peaks confirms formation of zinc oxide. It is also noticed that the sample contains a higher percentage of Zn and less percentage of oxygen. The EDAX template of Ni/Cu-ZnO NPs is represented in Fig. 8(b–d). In these spectra, characteristics Lα, Kα, and Kβ peaks at 1 KeV, 8.6, and 9.6 KeV indicate the presence of Zn. The purity of the synthesized Ni/Cu-ZnO has been confirmed by the presence of characteristics of Ni and Cu peaks. Table 1 gives the weight and atomic % of elements constituting the nickel and copper incorporated zinc oxide.

**Table 1:** Elemental composition of (a) un-doped ZnO (b) 10 % Ni_10_Cu_10_ZnO_80_ (c) 20 % Ni_20_Cu_20_ ZnO_60_ (d) 30 % Ni_30_Cu_30_ZnO_40_.

Sample	Element	Weight%	Atomic%
a) Un-doped ZnO	O K	13.44	83.82
	Zn K	86.56	61.18
	Total	100	
b)10 % Ni: Cu: ZnO	O K	42.73	74.86
	Ni K	9.01	4.3
	Cu K	11.58	5.11
	Zn K	36.68	15.73
	Total	100	
c)20 % Ni: Cu: ZnO	O K	44.71	76.15
	Ni K	13.06	6.06
	Cu K	14.71	6.31
	Zn K	27.52	11.47
	Total	100	
d) 30 % Ni: Cu: ZnO	O	13.03	36.90
	Ni	28.51	22.01
	Cu	28.15	20.08
	Zn	30.31	21.02
	Total	100	

### 6.1 Anticancer performance studies

**Fig. 9:**
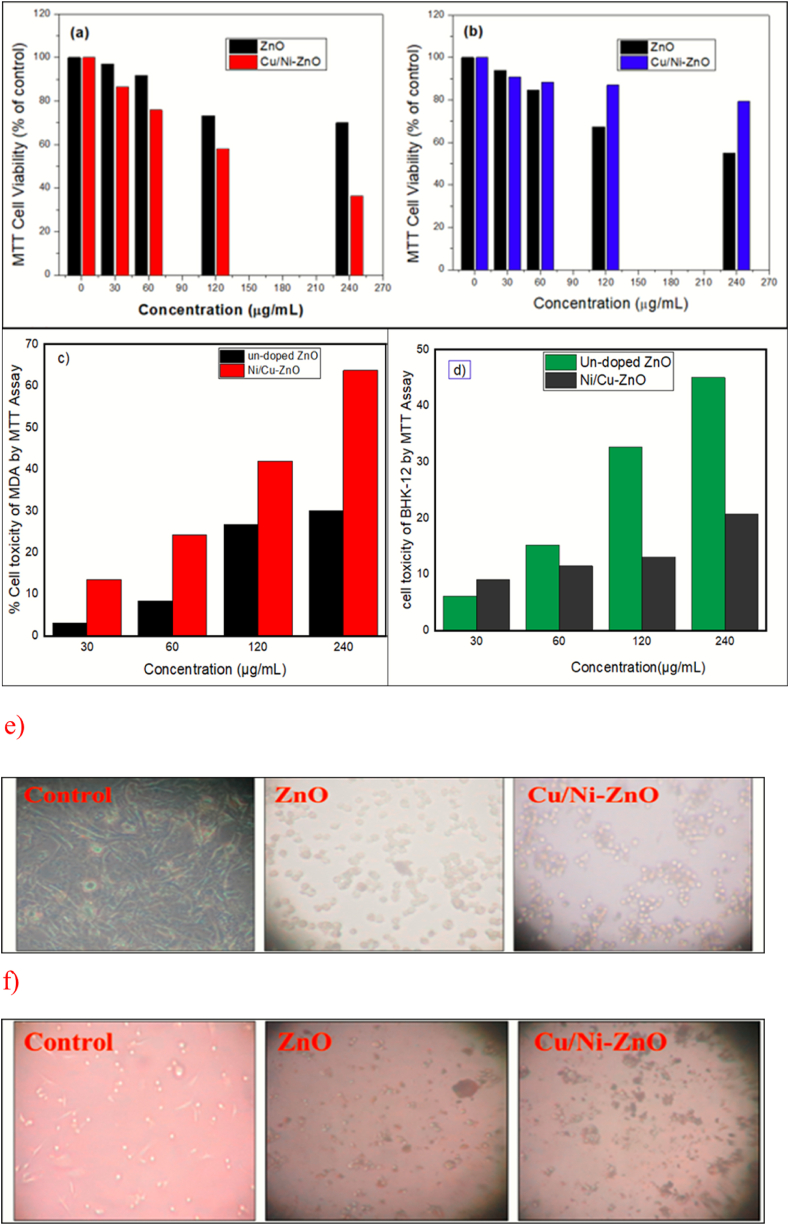
Anticancer activity of pure ZnO NPs, and Cu/Ni-ZnO NPs versus epithelial breast cancer (MDA) and kidney (BHK-21) skin cell lines. MTT assays showing the dosage dependence of % cell viability results of pure ZnO and Cu/Ni-ZnO NPs (a) MDA (b) BHK-21 cells. MTT assays showing the dosage dependence of % cell toxicity results of pure ZnO and Cu_20_Ni_20_ZnO_60_ NPs (c) MDA and (d) BHK-21 cells. (e) Morphological changes in MDA cells after the 24 h exposure to 120 μg/mL of ZnO and Cu/Ni-ZnO NPs. Whereas, (f) is the morphology change in the BHK-21 cells exposed to 120 μg/mL of pure ZnO and Ni_20_Cu_20_-ZnO_60_ NPs for 24 h.

ZnO NPs have shown intrinsic anticancer capabilities that can be further enhanced by tuning the physicochemical properties modification. The present work is designed to raise the anticancer activity of ZnO by incorporating Cu and Ni into the ZnO lattice to form a solid solution. The anticancer activity in the epithelial breast cancer cell (MDA) and kidney (BHK-21) skin cells was determined by exposing them for 24 h to the different concentrations (control, 30, 60, 120, and 240 μg/mL) of ZnO and Ni/Cu-ZnO NPs solid solution. MTT cell viability results show the dose-dependent of pure ZnO and Ni_20_Cu_20_ZnO_60_ NPs had a dosage range of 30–240 μg/mL Fig. 9(a–b).

It can be observed that as-prepared pure ZnO and Ni_20_Cu_20_ZnO_60_ NPs demonstrated strong cytotoxic effect for the tested cell line in comparison with the normal skin cell line. Furthermore, the acquired results showed that high cell mortality occurred at high concentrations of Ni/Cu-ZnO as compared with pure ZnO NPs. The result indicated the maximum cell growth inhibition of cancer cells was perceived at 240 μg/mL was 63.7 % by Ni/Cu-ZnO NPs. The result showed a relationship between % cell viability and % cell toxicity among the human breast cancer (MDA) and skin cell lines (BHK-21) respectively with many concentrations of NPs. As the concentration of the NPs increases, cell viability is reduced. The detected maximum decrease in the cell viability of MDA indicates the anticancer properties of Ni_20_Cu_20_ZnO_60_ NPs and pure ZnO. The Ni/Cu-ZnO NPs were more effective for the treatment of MDA and showed minimum % cell viability at a concentration of 240 μg/mL was 36.26 %, while at the same concentration, it showed 79.44 % for BHK-21. which concluded that Ni/Cu-ZnO was more effective for human breast cancer cell treatment and less harmful for normal skin cells BHK-21. The IC50 values against the MDA cell line were measured at 5.82 μg/mL for undoped QDs and 3.34 μg/mL for Ni/Cu-ZnO QDs.

The MDA and BHK-21 cell morphology after the exposure of 120 μg/mL of ZnO, Ni/Cu-ZnO NPs for 24 h is presented in Fig. 9(c–d). The pictures depicted in Figure (9 c) show that the significant cell deaths of MDA cells appear by the Ni/Cu-ZnO compared to ZnO and control. However, the BHK-21 cell death caused by the ZnO and Ni/Cu-ZnO NPs is lower compared to the MDA cell deaths. The results are in agreement with the MTT assay. The higher anticancer activity by the Ni/Cu-ZnO NPs could be due to the synergistic effect of Cu, Ni, and ZnO functional materials.

### 6.2 Antioxidant activity analysis

**Fig. 10:**
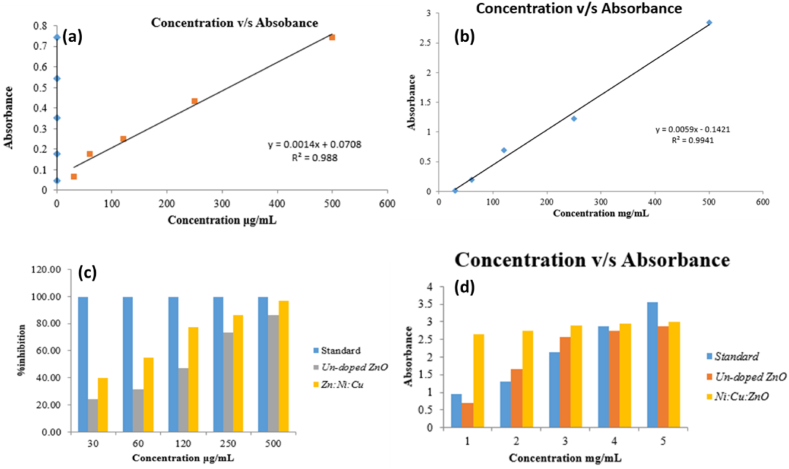
Illustrate the antioxidant activity of pure ZnO, and Ni/Cu-ZnO, and the results were assessed with the standard ascorbic acid under the same conditions. The antioxidant activity is determined by the total amount of antioxidants by (a) pure ZnO, (b) Ni/Cu-ZnO NPs, (c) Ferric Reducing antioxidant capacity, and (d) DPPH approach by pure ZnO and Ni/Cu-ZnO NPs.

The linear regression equation (y = 0.0014x+0.0708 with R^2^ = 0.988, where y was the absorbance of samples, intercept 0.0708, slope 0.0014, and ascorbic acid concentration per mg/mL was x) of the calibration curve was used to accomplish the TAC assay of pure ZnO and Ni/Cu- ZnO NPs. The TAC of pure ZnO (Fig. 5(a)) and Ni/Cu-ZnO Fig. 10 (b) was 0.549 and 1.932 g ascorbic acid equivalent/gram of NPs with various dilutions as shown in Fig. 10(a). The accurate capacity of TAC assessment was the development of phosphor molybdenum complexes. This technique comprises reducing complex molecules with synthesized NPs [50]. The antioxidant activity of pure ZnO and Ni/Cu-ZnO NPs was also calculated by the FRAP method. The method includes the reduction of ferric ions to ferrous ions [50]. The calibration curve's ferric reducing antioxidant capacity was determined using a linear regression line y = 0.0059x + 0.1421 in which x = ascorbic acid concentration in mg/mL, and y = absorbance with R^2^ = 0.9941 Fig. 10(c). Pure ZnO and Ni/Cu-ZnO had antioxidant activities of 81.78 and 84.94 mg of ascorbic acid/gram of NPs, respectively as shown in Table 2. The method of applying free reducing acid power is relatively effective [51,52].

**Table 2:** Determination of total antioxidant activity.

parameter	Regression line	Quantitative value(g ascorbic acid + NPs extraction equivalent/g)	
		Pure ZnO	Ni/Cu-ZnO
Total antioxidant capacity	y = 0.0014x+0.0708	0.549	1.932
Ferric reducing antioxidant power	y = 0.0059X-0.1421	81.78 mg	84.94 mg

Another precise and efficient methodology, DPPH, can also be used to find out the antioxidant activity of NPs [53]. As the appearance of the final reaction mixture shifted from purple to yellow due to the take on hydrogen atoms by DPPH, maximum absorption was seen at 517 nm, as shown in Fig. 10(d). Detecting the antioxidant activity of substances at lower concentrations is simple and quick [54]. Table 3 displays the ascorbic acid reference values together with the IC50 values for undoped and Ni/Cu-ZnO NPs at different doses as determined by the DPPH radical scavenging experiment. When it came to Ni/Cu-ZnO NPs, the concentration at which the lowest IC50 value (0.863 μg/mL) observed at 30 μg/mL, while the concentration at which the greatest IC50 value (4.71 μg/mL) was recorded at 500 μg/mL. Comparatively, pure ZnO showed the greatest IC50 value of 14.61 μg/mL at 500 μg/mL and the lowest IC50 value of 4.11 μg/mL at 30 μg/mL concentration. Notably, when the concentration of Ni/Cu-ZnO nanoparticles increased, so did the DPPH free radical scavenging capacity.

**Table 3:** The DPPH assay antioxidant activity of undoped ZnO and Ni_20_Cu_20_ZnO_60_.

Concentration (μg/mL)	Un-doped ZnO	Ag_20_Co_20_ZnO_60_	Standard	IC 50 (μg/mL)
30	24.11	39.94	99.61	0.863
60	31.29	54.87	99.66	1.888
120	47.29	77.05	99.76	3.410
250	73.19	86.50	99.85	4.058
500	86.11	96.99	99.90	4.778

**Fig. 11:**
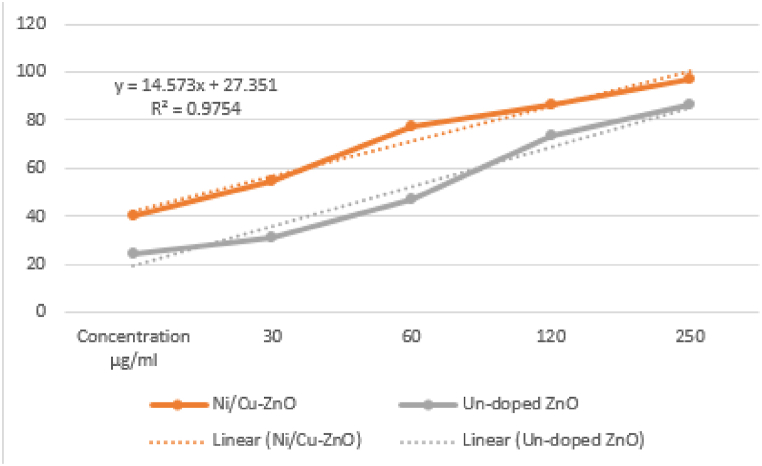
Reducing power of pure ZnO and Ni/Cu-ZnO NPs.

The reducing power (RP) quantifies the deactivation of oxidants by linking to redox processes. The chemicals' ability to decrease other compounds is an important indication of their potential antioxidant effect. High chemical absorbance, as demonstrated in the case of synthesized NPs, is an indication of enhanced reducing power [55]. The Ni/Cu-ZnO NPs show higher reducing power than ZnO NPs, as appeared in Fig. 11, and the results are tabulated in Table 4.

**Table 4:** The reduction power of undoped ZnO and Ni_20_Cu_20_-ZnO_60_.

Concentration (μg/mL)	Un-doped ZnO	20 % Ni/Cu-ZnO	Standard
30	0.6907	2.6543	0.958
60	1.6642	2.7536	1.294
120	2.5544	2.8931	2.135
250	2.7405	2.9578	2.894
500	2.8694	2.9936	3.562

### 6.3 Molecular docking

**Antioxidant study for MDA**: Molecular docking analysis of Ni/Cu-ZnO was performed to get an insight into mechanistic interactions with target enzymes, human peroxiredoxin 5. The importance of these enzymes lies in their role in the direct elimination of H_2_O_2_ and the neutralization of other reactive oxygen species (ROS). Illustrated in Fig. 4(b) is the optimal docking conformation of Ni/Cu-ZnO with human peroxiredoxin 5, exhibiting a binding score of −4.3 kcal mol^−1^. Ni/Cu-ZnO demonstrated hydrogen bonding connections with crucial amino acid deposits such as GLU16, GLY83, and LYS 93 within the active pocket, in addition to metal interactions, suggesting the potential of Doped ZnO as an inhibitor of human peroxiredoxin 5, as shown in Fig. 4(b). All these docking results are in conformity with experimental work as mentioned earlier. The Binding score of undoped and doped ZnO against β human peroxiredoxin 5 is given in Table 5.

**Table 5:** Molecular docking results of undoped and doped ZnO against human peroxiredoxin 5.

Ligand	Protein PDB ID	Docking Score
		kcal mol^−1^
ZnO	1HD2	−3.9
Ni/Cu doped ZnO		−4.3

### 6.4 Anticancer study for MDA

Molecular docking analysis of Ni/Cu- ZnO was performed to get an insight into mechanistic interactions with target enzymes. Estrogen receptor alpha (ERα; PDB: 5GS4) is of significant importance in the context of the progress and development of hormonal-dependent type breast cancer. The docking analysis revealed an optimal conformation (binding score = −4.2 kcal mol^−1^) of Ni_20_Cu_20_ZnO_60_ with estrogen receptor alpha, as illustrated in Fig. 3(c). Notably, Ni/Cu-ZnO exhibited hydrogen bonding interactions with critical amino acid residues such as GLU 353, MET 357, and LEU 387 within the active pocket, in conjunction with metal contact interactions. These findings suggest the potential of Ni/Cu-ZnO as an inhibitor targeting the estrogen receptor alpha enzyme. All these docking results are in conformity with experimental work as mentioned earlier. The Binding score of undoped and bimetallic doped ZnO against estrogen receptor alpha Erα target is given in Table 6.

**Table 6:** Molecular docking results of undoped and Ni/Cu-ZnO against estrogen receptor alpha Erα.

Ligand	Protein PDB ID	Docking Score
ZnO	5GS4	−3.8
Ni/Cu-ZnO		−4.2

**Antioxidant study for BHK-21**: Molecular docking analysis of Ni_20_Cu_20_ZnO_60_ was performed to get an insight into mechanistic interactions with target enzymes, human peroxiredoxin 5. The importance of these enzymes lies in their role in the direct elimination of H_2_O_2_ and the neutralization of other reactive oxygen species (ROS). The most favorable binding conformation (with a binding score of −4.3 kcal mol^−1^) of the Ni_20_Cu_20_ZnO_60_ complex through human peroxiredoxin 5 is illustrated in Fig. 4(b). Within the active pocket, Ni/Cu-ZnO exhibited hydrogen bonding contacts with key amino acid remainders such as GLU16, GLY83, and LYS93, in addition to metal interactions, thereby suggesting that transition metal-doped ZnO could serve as a promising inhibitor of human peroxiredoxin 5, as shown in Fig. 4(b). All these docking results are in conformity with experimental work as mentioned earlier. The Binding score of undoped and doped ZnO against β human peroxiredoxin 5 is given in Table 7.

**Table 7:** Molecular docking results of undoped and doped ZnO against human peroxiredoxin 5.

Ligand	Protein PDB ID	Docking Score
		kcal mol^−1^
ZnO	1HD_2_	−3.9
Ni/Cu-ZnO		−4.3

**Anticancer study for BHK-21**: Molecular docking analysis of Ni/Cu-ZnO was performed to get an insight into mechanistic interactions with target enzymes, estrogen receptor alpha (ERα; PDB: 5GS4) and the protein kinase Akt target [PDB:7NH5] for BHK-21 normal skin cell lines. The essentiality of these enzymes for the development and advancement of hormonal-dependent type breast cancer and Akt1 (Protein Kinase) is a key regulator of cell survival, metabolism, and proliferation. The optimal docked conformation (with a binding score of −4.2 kcal mol^−1^) of Ni_20_Cu_20_-ZnO_60_ interacting with estrogen receptor alpha is illustrated in Fig. 5(c). Ni/Cu-ZnO exhibited hydrogen bonding interactions with crucial amino acid residues such as GLU 353, MET 357, and LEU 387 within the active pocket, in addition to metal-contact interactions, indicating the potential of Doped ZnO as an inhibitor for the specific enzyme. Furthermore, the association of Ni/Cu-ZnO with the active pocket of the protein kinase Akt1 target is linked to a metal site and hydrogen bonding interactions with GLN 79, TRP 80, ASP 274, ASP 292, and PHE 293, resulting in a binding score of −5.0 kcal mol-1, as represented in Fig. 5(d). All these docking results are in conformity with experimental work as mentioned earlier. The Binding score of undoped and Ni/Cu-ZnO against estrogen receptor alpha Erα and the protein kinase Akt1 target is given in Table 8.

**Table 8:** Molecular docking results of undoped and Ni/Cu-ZnO against estrogen receptor alpha Erα and protein kinase Akt1.

Ligand	Protein PDB ID	Docking Score
ZnO	5GS4	−3.8
Ni/Cu doped ZnO		−4.2
ZnO	7NH5	−4.3
Ni/Cu doped ZnO		−5.0

## 7 Conclusions

In conclusion, the study successfully synthesized Ni/Cu-ZnO NPs, confirming their purity and chemical composition through EDX analysis. The anticancer activity of these NPs was assessed on MDA breast tumor and BHK-21 skin cell lines, revealing a dose-dependent cytotoxicity and enhanced efficacy of Ni/Cu-ZnO compared to pure ZnO. Morphological changes in cell cultures supported the MTT assay results, indicating the superior anticancer activity of Ni/Cu-ZnO, possibly due to the synergistic effects of Ni, Cu, and ZnO materials. Additionally, antioxidant activity analysis using TAC, DPPH, and FRAP assays demonstrated the enhanced antioxidant properties of Ni/Cu-ZnO compared to pure ZnO NPs. The reducing power assay further highlighted the superior antioxidant potential of Ni/Cu-ZnO. These judgments suggest the promising potential of Ni/Cu-ZnO NPs for both anticancer and antioxidant applications. But still, the reproducibility and scalability of NPs need to be addressed. The current study synthesis method shows promise, it must be optimizing at bulk production for clinical application to ensure cost-effectiveness and consistency. while in vitro studies are indispensable for preliminary assessments, the incorporation of in vivo information is essential to enhance the thoroughness and precision of evaluating nanoparticles' capabilities.

Molecular docking studies were conducted to evaluate the antioxidant and anti-cancer properties of undoped ZnO and Ni/Cu-ZnO NPs. The molecular docking was carried out on the active site of human peroxiredoxin 5 (PDB code 1HD2, alpha estrogen receptor alpha (ERα; PDB: 5GS4) and pirin inhibiting target [PDB: 3ACL] for antioxidant study, breast cancer and skin cancer cell lines respectively using Auto dock vina program. The docking score of the Ni/Cu-ZnO NPs into the active pockets of target receptors suggested the produced bi-metallic doped ZnO as a promising inhibitor of these enzymes.

## CRediT authorship contribution statement

**Huma Ayub:** Investigation, Methodology, Writing – original draft. **Uzma Jabeen:** Data curation, Formal analysis, Software, Validation. **Iqbal Ahmad:** Conceptualization, Project administration, Resources, Supervision. **Muhammad Aamir:** Writing – review & editing. **Asad Ullah:** Formal analysis, Visualization. **Ayesha Mushtaq:** Formal analysis, Methodology. **Farida Behlil:** Data curation, Formal analysis. **Binish Javaid:** Formal analysis, Investigation. **Asad Syed:** Formal analysis, Resources. **Abdallah M. Elgorban:** Formal analysis, Resources. **Ali H. Bahkali:** Formal analysis, Resources. **Rustem Zairov:** Formal analysis, Funding acquisition, Validation. **Asad Ali:** Formal analysis, Funding acquisition, Methodology, Writing – review & editing.

## Declaration of competing interest

The authors declare that they have no known competing financial interests or personal relationships that could have appeared to influence the work reported in this paper.
